# Agents Strongly Preferred: ERP Evidence from Natives and Non-Natives Processing Intransitive Sentences in Spanish

**DOI:** 10.3390/brainsci12070853

**Published:** 2022-06-29

**Authors:** Adam Zawiszewski, Gillen Martinez de la Hidalga, Itziar Laka

**Affiliations:** 1 Department of Linguistics and Basque Studies, University of the Basque Country (UPV/EHU), 01006 Vitoria-Gasteiz, Spain; itziar.laka@ehu.eus; 2 EPA-HHI Arrasateko Helduen Eskola, 20500 Arrasate, Spain; gillenmtz@gmail.com

**Keywords:** intransitive sentence processing, ERPs, agents, patients, non-native language processing

## Abstract

Are non-native speakers able to process their second language in a native-like way? The present study used the Event-Related Potentials’ (ERPs) method to address this issue by focusing (1) on agent vs. agentless intransitive sentences and (2) on person vs. number agreement morphology. For that purpose, native and high proficiency and early non-native speakers of Spanish were tested while processing intransitive sentences containing grammatical and ungrammatical subject–verb agreement. Results reveal greater accuracy in the agent (unergative) condition as compared with the agentless (unaccusative) condition and different ERP patterns for both types of verbs in all participants, suggesting a larger processing cost for the agentless sentences than for the agentive ones. These effects were more pronounced in the native group as compared with the non-native one in the early time window (300–500 ms). Differences between person and number agreement processing were also found at both behavioral and electrophysiological levels, indicating that those morphological features are distinctively processed. Importantly, this pattern of results held for both native and non-native speakers, thus suggesting that native-like competence is attainable given early Age of Acquisition (AoA), frequent use and high proficiency.

## 1. Introduction

The growing scientific interest in bilingualism during the last decades has raised many questions (though not always answers) about language learning and processing; in particular, it has generated new evidence about how the two languages of the bilingual interact and are processed and controlled. This body of evidence strongly suggests that learning a second language is “harder” than learning a first language even when exposure to the second language is early and frequent [1,2,3,4,5] so that native competence might be difficult to achieve in a second language. Thus, it looks as if some aspects of the second non-native grammar can be learned and processed as natives do, while others cannot (see [6], for an overview). Here, we focus on the Language Distance Hypothesis [7]. This hypothesis claims that early and proficient bilinguals can achieve native competence for grammatical properties shared by their two languages, whereas unshared grammatical properties pose a challenge for native-like syntactic processing. We present a novel behavioral and Event-Related Potential (ERP) study where early and proficient Basque-Spanish bilinguals behave native-like in their second language, Spanish, when processing (a) argument structure alternations in intransitive sentences involving agent vs. patient subjects and (b) subject–verb agreement, both of which are grammatical properties shared by the two languages of these bilinguals.

ERPs are a very reliable tool to determine the time course of language-related brain processes occurring at the electrophysiological level (see [8], for an overview). In general, three main types of language-related ERP components have been reported in the literature: the Left Anterior Negativity (LAN), the N400, and the P600. LAN is a negative deflection of the wave distributed over the left anterior sites of the scalp which appears between 300 and 500 ms after stimulus onset. It has been described as a response to syntactic and morphological manipulations, most commonly to agreement violations (see [9], for an overview and discussion). On the other hand, the N400 is a negative deflection of the wave with a centro-parietal distribution peaking in amplitude between 300 and 500 ms after stimulus presentation regularly reported as a response to semantic plausibility violations (see [10], for an overview), anomalous thematic hierarchy [11], or less frequent and unexpected words [12]. Lastly, the P600 is a positive-going wave distributed over central and parietal electrodes starting around 500 ms after stimulus onset and lasting about 300 ms. It has been usually interpreted in terms of reanalysis or integration mechanisms occurring when syntactically complex, ill-formed or ambiguous information is being processed (see [13]; for discussion). Besides, many studies have reported more complex patterns as a response to morphosyntactic anomalies: LAN followed by a P600 (see [14], for an overview) or an N400 followed by a P600 [5,15].

Previous studies focusing on non-native processing have successfully identified some relevant factors it is modulated by, namely Age of Acquisition (AoA) (e.g., [14]), level of proficiency/use ([16], among others) and the degree of similarity between the native (L1) and non-native languages (L2) (e.g., [12]).

Weber-Fox and Neville [4], for instance, tested Chinese–English adult bilinguals exposed to English at different ages during their life span who were required to read grammatical and ungrammatical sentences. Results showed differences between English monolinguals and L2 speakers regarding syntactic processing at both behavioral and electrophysiological levels (i.e., longer latencies and lack of early negativity in the latter group), and those differences emerged even in those participants whose exposure to English was delayed for 1–3 years from birth. According to the authors, these findings indicate that maturational changes constrain the development of neuronal systems relevant for language and that different components display different sensitive periods (syntax being more sensitive than semantics). On the other hand, Rossi and collaborators [16] investigated highly proficient and late (AoA > 10 years) Italian–German and German–Italian speakers when listening to sentences containing word category and subject–verb agreement violations in their respective second languages. Results revealed that, despite the relatively late AoA onset, non-native speakers displayed a similar ERP pattern to that of natives (early LAN—ELAN—for category violations and LAN for agreement violations, both followed by a P600), indicating that at high proficiency, non-native speakers can perform in a native-like way. Finally, Zawiszewski et al. [5] showed that on top of AoA and proficiency, the degree of similarity between the native and non-native grammars (labeled by the authors as *language distance*) also plays a significant role in L2 processing. They examined how Basque–Spanish and high-proficiency and early (AoA = 3 years) Spanish–Basque speakers process subject–verb agreement and case morphology in Basque. Importantly, Basque and Spanish grammars both have subject–verb agreement and are thus similar in that grammatical trait, but fundamentally diverge with respect to case marking (Basque is an ergative language that overtly marks agents regardless of transitivity, while Spanish is nominative, lacking agent-specific case morphology). Results revealed a similar ERP pattern in both groups of speakers in the verb agreement condition (in comparison to grammatical agreement, the violations elicited an N400 followed by a P600), but a different one in the case morphology condition (an N400 followed by a P600 in natives and an N400 in the non-native group). Based on this evidence, the authors concluded that language distance has a significant modulating effect on non-native language processing.

Intransitive predicates in language form two main groups regarding the nature of their subjects: (a) those with an agent, such as *laugh* (e.g., *Mary laughed at her jokes*), and (b) those with a patient, such as *fall* (e.g., *Mary fell in the river*). A significant body of research on sentence processing [8,17] argues that there is a universal preference for interpreting sentence-initial NPs as agents, that is, there is a cross-linguistic preference for agent subjects (see also [18,19], for an overview of the literature on agent-first preference). This predilection for interpreting the initial argument as an agent has also been found among young children. More precisely, Abbot-Smith and collaborators [20] investigated, in an eye-tracking study, whether two-year-old (group 1) and three-year-old (group 2) children use a first-NP-as-agent bias to process active transitive and passive sentences. The results showed that both age groups showed a tendency to map the first noun in a sentence onto an agent role. The assumption that agent-first arguments are preferred over patient-first arguments has been explicitly formulated within the extended argument dependency model (eADM) [17]. This proposal assumes two main grammatical roles, namely the actor and the undergoer, which correspond to the agent and patient prototypes, respectively. According to the authors, the assignment of a specific role depends on the particular language being processed: while in some languages (e.g., English) argument position is a reliable cue, in others the relevant information is provided by morphology (e.g., German, Japanese). The predictions made by the eADM have been largely corroborated by experimental data. For instance, ERP evidence from German [11] has revealed that processing inanimate agents is costlier than processing animate ones, that is, in the sentence “Paul asks himself [which teacher]_ACC_ [the twig]_NOM_ brushed” processing *the twig* is costlier in comparison with an animate NP in the same position, and this cost is reflected by an N400 component. This effect is presumably due to the increased processing cost associated with having to assign an actor role to an inanimate argument. In another ERP study carried out in Turkish, Demiral, Schlesewsky and Bornkessel-Schlesewsky [21] showed an increased processing difficulty (P600) when an initial ambiguous argument was disambiguated towards an object (=patient/theme) reading, as compared with agent subjects. In a similar vein, Bickel et al. [22] used ERPs to investigate agent vs. patient preference in Hindi and showed a clear agent-first preference when processing the initial arguments, that is, disambiguation of an initial NP towards a patient reading engendered a biphasic N400—P600 pattern. In sum, experimental evidence presented so far suggests that subjects that bear a patient relation to the verb (as in *the child fell*) will incur larger processing costs than subjects that bear an agent relation to the verb (as in *the child laughed*).

Regarding the second aspect to be examined in the present study, namely, the phi-features person and number, only a few experimental works have investigated and compared their processing by means of ERPs. Silva-Pereyra and Carreiras [23] compared person, number and person + number violations in Spanish, and a similar Anterior Negativity followed by a P600 emerged for person and number. On the other hand, Mancini et al. [24] reported an N400 + P600 pattern for person violations, and an LAN + P600 pattern for number violations in Spanish. According to the authors an N400 effect is expected for violations that have an impact at the interface with the semantic–discourse representation of the sentence (person violations); while a LAN effect is expected for violations limited within the boundary of the morphosyntactic representation (number violations). Person and number features have also been tested in other languages, and this LAN/N400 difference has not been reported elsewhere. Zawiszewski et al. [25] compared person, number and person + number violations in Basque, and found an N400 followed by a P600 for all types of violations; however, person violations and combined person + number violations yielded a larger P600 than number violations, suggesting that person is more salient than number in terms of phi-features (see [26], for a more detailed description of person and number feature processing).

## 2. The Present Study

We examine these two dimensions of subjecthood in non-native language processing: (a) agent preference and (b) person/number agreement by means of ERPs in order to compare how native speakers of Spanish and native speakers of Basque who were highly proficient speakers of Spanish with an early AoA process (a) agent vs. patient intransitive subjects and (b) person vs. number agreement features in Spanish. The purpose of this comparison is to determine whether these early and proficient non-natives are native-like in their processing profiles. Spanish is a Subject Verb Object (SVO), nominative-accusative language where subjects carry nominative case, regardless of their semantic role; thus, besides prototypical agent subjects such as *la niña agarra el juguete* (the child grabs the toy), there are also patient subjects like *la niña nació ayer* (the child was born yesterday). On the other hand, Basque is a Subject Object Verb (SOV), ergative language where agent subjects bear ergative case (e.g., *umeak jostailua hartzen du* ‘the child grabs the toy’) and patient subjects are morphologically unmarked (e.g., *umea atzo jaio zen* ‘the child was born yesterday’) [27]. Since Spanish and Basque mark agent and patient subjects differently, our aim was to test the potential impact of these typological differences on SV agreement processing in native and non-native speakers. In other words, in Spanish all intransitive subjects are morphologically unmarked (nominative case) while in Basque intransitive agents carry an ergative marker (-k) and intransitive patients are unmarked (absolutive case). Previous studies on ergative morphology and non-native language processing (e.g., [5,7]) have revealed that typological differences between the L1 and L2 are an aspect that non-native speakers are particularly sensitive to; therefore, investigating SV agreement processing in native and non-native speakers of Spanish will allow us to look into this issue in more detail.

### 2.1. Hypotheses

In this study we use grammatical and ungrammatical subject–verb agreement dependencies in order to test the agent preference and the Feature Distinctness Hypothesis (FDH) ([28]; see also [24]) in native and non-native speakers of Spanish. Given that in both Spanish and Basque (the native language of the non-native group) the verb agrees overtly with the subject, and, given the high proficiency and low AoA of the L2 speakers, according to the Language Distance Hypothesis [7], similar behavioral and electrophysiological responses to agreement violations are expected for all participants. Since the agent-first hypothesis has not been previously tested in intransitive sentences and nominative-accusative languages by means of ERPs, and based on previous evidence and literature regarding SV agreement (see [29], for an overview), we tentatively predict that SV violations will elicit a negative component (LAN or N400) followed by a P600. According to the agent-first hypothesis, a larger processing cost should be observed for sentences containing patient subject predicates than for those containing agent subject verbs. Similarly, according to previous work on phi-feature processing [24,25] differences between person and number feature processing should emerge as well, namely, person violations should elicit a larger P600 in comparison to the number violations. In addition, we might also find a negative component as a response to these violations (LAN/N400).

### 2.2. Participants

Twenty-seven native (10 men; mean age 20.5 years, SD = 2.9) and 25 non-native (5 men; mean age 21.8 years, SD = 2.9; AoA = 5.7 years, SD = 1.9) speakers of Spanish took part in the experiment. According to the Edinburgh Handedness inventory [30], all participants were right-handed and they were all paid for their participation (see Table 1 for details). Data from 3 native and 1 non-native participants were removed due to the insufficient number of segments available for statistical analyses. The study was approved by the Ethics Committee of the University of the Basque Country (UPV/EHU)) (M10_2020_182).

### 2.3. Materials

Two hundred fifty-six experimental sentences and 160 fillers (416 in total) were created and distributed over 4 counterbalanced lists. The materials were organized according to a 2 × 2 × 2 design: subject type (agent vs. patient), feature (person and number), and grammaticality (grammatical and ungrammatical) (see Table 2). The design of the materials was motivated by the assumption that 1st and 2nd person singular forms have a specification for the person feature, but not for number, while 3rd person is specified for number, but not for person [31,32]. Consequently, we followed the design used in Mancini et al. [24] and Martinez de la Hidalga et al. [28,33]. More precisely, for person conditions 2nd person was used in the grammatical condition and 1st person was used in the ungrammatical manipulation. For number conditions, 3rd singular vs. 3rd plural manipulations were used. A distance between subjects and verbs was created by adding three words and an average of 2.6 words (1, 2, or 3 words) were added after the critical word, controlled per condition. The materials were controlled for length and frequency.

### 2.4. Procedure

Personal computers (Windows 7 operating system) and Presentation software (version 16.3) were used to present the stimuli on the screen. Before the actual experiment began, participants were instructed about the EEG procedure and seated comfortably in a quiet room in front of a 24 in. monitor. The experiment was conducted in a silent room in the Experimental Linguistics Laboratory at the University of the Basque Country (UPV/EHU) in Vitoria-Gasteiz. Sentences were displayed in the middle of the screen word by word for 350 ms (ISI = 250). A fixation cross (+) indicated the beginning of each sentence trial. After each trial the words *correcto?* “correct?” or *incorrecto?* “incorrect?” appeared in the screen, and participants were asked to judge the acceptability of the previously displayed sentence as either correct (left Ctrl) or incorrect (right Intro). Half of the participants used the left hand for correct responses and the other half the right hand. All 416 sentences were distributed randomly in four blocks that lasted approximately 10 min each. Participants had a short break between each block which lasted as long as they needed. Before the actual experiment, participants ran a short training session of three trials. They were asked to avoid blinking or moving when the sentences were being displayed and to make the acceptability judgment as fast and accurate as possible. The whole experiment, including electrode-cap application and removal, lasted about 1h15m.

### 2.5. EEG Recording

The EEG was recorded from 32 active electrodes secured in an elastic cap (Acticap System, Brain Products). Electrodes were placed on standard positions according to the extended Internationals 10–20 system in the following sites: Fp1/Fp2, Fz, F3/F4, F7/F8, FC5/FC6, FC1/FC2, T7/T8, C3/C4, Cz, CP5/CP6, CP1/CP2, P7/P8, P3/P4, Pz, O1/02, Oz, LM, VEOG and HEOG. All recordings were referenced to right mastoid position and re-referenced off-line to the linked mastoids. Vertical and horizontal eye movements and blinks were monitored by means of two electrodes positioned beneath and to the right of the right eye. Electrode impedance was kept below 5 kOhm for all scalp electrodes and below 10 kOhm for the eye electrodes. The electrical signals were digitized online at a rate of 500 Hz by a Brain Vision amplifier system and filtered offline within a band pass of 0.1–35 Hz. After the EEG data were recorded, the ocular correction procedure [34] as well as the artifact rejection procedure were applied (offline). Trials with other artifacts were removed when they indicated any voltage exceeding 150 μV and voltage steps between two sampling points exceeding 35 μV.

### 2.6. Data Analysis

For the data analysis, the following types of subject agreement structures were compared: agent subject with the grammatical and ungrammatical person (*actuarás* ‘play.2SG.FUT.’ vs. *actuaré ‘play.1SG.FUT.’; conditions 1 vs. 2 in Table 2); agent subject with the grammatical and ungrammatical number (*actuará* ‘play.3SG.FUT.’ vs. *actuarán ‘play.3PL.FUT’; conditions 3 vs. 4 in Table 2, respectively); patient subject with the grammatical and ungrammatical person (*vendrás* ‘visit.2SG.FUT.’ vs. *vendré ‘visit.1SG.FUT.’; conditions 5 vs. 6 in Table 2); patient subject with the grammatical and ungrammatical number (*vendrá* ‘visit.3SG.FUT.’ vs. *vendrán ‘visit.3PL.FUT.’; conditions 7 vs. 8 in Table 2, respectively). For the ERP measures, segments were created starting at 200 ms before and ending 1000 ms after the onset of the critical words (the verb) in the sentences. The trials associated with each subject type were averaged for each participant. The EEG 200 ms prior to the onset was also used as a baseline for all sentence type comparisons. After visualizing the data and based on the literature, 300–500 ms and 600–900 ms temporal windows were considered during statistical analysis in all conditions. After the stimuli were recorded and averaged, analyses of variance (ANOVA) were carried out in nine regions of interest that were computed out of 27 electrodes: lateral electrodes: left frontal (F7, F3, FC5), left central (T7, FP5, C3), left parietal (P7, P3, O1), right frontal (F4, F8, FC6), right central (C4, FP6, T8), and right parietal (P8, P4, O2); midline electrodes: frontal (Fp1, Fz, Fp2), central (FC1, Cz, FC2), and parietal (CP1, Pz, CP2). Repeated-measures ANOVAs were performed in all experimental manipulations and trials (correctly and incorrectly judged trials) for each window of time using five within-subjects factors: grammaticality (2 levels: grammatical, ungrammatical), subject type (2 levels: agent, patient), agreement feature (2 levels: person, number), hemisphere (2 levels: left, right), and region (3 levels: frontal, central and parietal). Midline (frontal, central, and parietal) electrodes were analyzed independently. Whenever the sphericity of variance was violated, a correction [35] was applied to all the data with greater than one degree of freedom in the numerator. Finally, further statistical comparisons were conducted (split by the grammaticality condition) whenever an interaction turned out to be statistically significant. Effects for subject type, agreement feature, hemisphere or region factors are only reported here when they interacted with the experimental manipulation of grammaticality.

For the behavioral results, error rates and response latencies of all the trials were submitted to repeated measures ANOVAs with grammaticality (two levels: grammatical, ungrammatical), subject type (two levels: agent, patient) and feature (two levels: person, number) conditions as within-subject factors. Subsequent comparisons (by subject and by item) were carried out whenever a grammatical interaction was significant.

## 3. Results

### 3.1. Behavioral Results

Regarding accuracy (see Table 3 and Table 4 for details), a GROUP effect was found (F1(1,46) = 4.68, *p* = 0.036; F2(1,508) = 69.15, *p* < 0.001), showing that overall natives were more accurate than non-natives (93.25% vs. 89.15%). The main TYPE effect was also found (F(1,46) = 6.73, *p* = 0.013; F2(1,508) = 3.71, *p* = 0.055), showing higher accuracy for sentences containing agent subjects (91.78%) than for sentences containing patient subjects (90.61%). The main GRAMMATICALITY effect (F1(1,46) = 16.78, *p* < 0.001; F2(1,508) = 131.6, *p* < 0.001) indicates that participants were more accurate when making their judgments about the grammatical sentences (94.32%) as compared with the ungrammatical sentences (88.07%). The main FEATURE effect turned out to be significant in the analysis by item (F2(1,508) = 7.88, *p* = 0.005), indicating that participants performed the task more accurately for sentences containing number feature manipulations (92.02%), than for sentences containing person feature manipulations (90.37%). A GRAMMATICALITY*GROUP interaction (in the analysis by item) (F1(1,46) = 2.18, *p* = 0.147; F2(1,508) = 16.94, *p* < 0.001) suggested that both groups were more accurate for the grammatical sentences than for the ungrammatical sentences (95.25% vs. 91.18%; 93.13% vs. 84.83%) (natives: F2(1,508) = 28.59, *p* < 0.001; non-natives: F2(1,508) = 118.96, *p* < 0.001). The comparison between both groups of participants revealed that natives were more accurate than non-natives both for the grammatical sentences (95.25% vs. 93.13%) (F2(1,508) = 0.69, *p* = 0.002), and for the ungrammatical sentences (91.18% vs. 84.83%) (F2(1,508) = 0.33, *p* < 0.001). A GRAMMATICALITY*FEATURE interaction F1(1,46) = 5.22, *p* = 0.027; F2(1,508) = 30.89, *p* < 0.001) indicated that participants were more accurate for grammatical sentences (94.81%) than for sentences containing person feature violations (85.94%) (F(1,46) = 13.84, *p* = 0.001), and similarly, participants were more accurate when making their judgments about the grammatical sentences (93.84%) than when deciding on sentences containing number feature violations (90.2%) (F(1,46) = 7.31, *p* = 0.009). The analysis by feature factor revealed that participants were slightly more accurate for number feature violations (90.2%) than for person feature violations (85.94%) (F(1,46) = 3.47, *p* = 0.069). A TYPE*GRAMMATICALITY*FEATURE interaction emerged in the analysis by item (F1(1,46) = 3.31, *p* = 0.075; F2(1,508) = 12.28, *p* < 0.001). The analysis by grammaticality factor showed that participants performed the task more accurately for grammatical patient subject sentences (93.09%) combined with person feature than for the cases where patient subjects combined with person feature violations (85.61%) (F(1,46) = 8.46, *p* = 0.006). Participants were also more accurate for grammatical patient subject sentences (93.75%) than for similar sentences containing number feature violations (89.98%) (F(1,46) = 6.15, *p* = 0.017). Participants also showed higher accuracy for grammatical agent subject sentences combined with person feature (96.52%) than for their equivalents containing person feature violations (86.26%) (F(1,46) = 19.49, *p* < 0.001). Similarly, participants were more accurate in the condition containing grammatical agentive sentences combined with number feature (93.93%) than in the equivalent condition containing number feature violations (90.43%) (F(1,46) = 6.15, *p* = 0.017). The analysis by type showed that participants were more accurate in the condition containing grammatical agents subject sentences with person feature manipulations (96.52%) than in the condition containing patient subject sentences with person feature manipulations (93.09%) (F(1,46) = 23.1, *p* < 0.001). The analysis by feature revealed that participants were slightly more accurate for patient subject sentences containing number feature violations (89.98%) than for patient subject sentences containing person feature violations (85.61%) (F(1,46) = 3.28, *p* = 0.077). Also, participants showed higher accuracy for grammatical agent subject sentences containing person feature manipulations (96.52%) than for grammatical agent subject sentences containing number feature manipulations (%93.75) (F(1,46) = 7.59, *p* = 0.008). On the other hand, participants were slightly more accurate when performing the task in the condition containing agent subject sentences with number violations (90.43%) than when dealing with agent subject sentences containing person violations (86.26%) (F(1,46) = 3.19, *p* = 0.08).

With regard to reaction times, a main GRAMMATICALITY effect (F1(1,48) = 19.98, *p* < 0.001; F2(1,508) = 201.64, *p* < 0.001) was found, indicating that participants reacted faster to ungrammatical sentences (626.29 ms) than to grammatical sentences (731.37 ms). A main GROUP effect emerged in the analysis by item (F2(1,508) = 3.98, *p* = 0.047), indicating that natives responded faster to the stimuli than non-natives (671.37 ms vs. 692.22 ms). A marginally significant TYPE*GRAM*GROUP interaction (F1(1,48) = 3.6, *p* = 0.064; F2(1,508) = 3.8, *p* = 0.052) revealed that participants reacted faster to sentences containing patient subject violations than to the corresponding grammatical sentences (natives: F(1,46) = 16.76, *p* < 0.001; non-natives: F(1,46) = 5.49, *p* = 0.023). Similarly, participants reacted faster to agent subject violations than to the corresponding grammatical sentences (natives: F(1,46) = 6.29, *p* = 0.016; non-natives: F(1,46) = 8.48, *p* = 0.006). Further analyses by type or group factors yielded no significant results. A GRAMMATICALITY*FEATURE interaction (F1(1,48) = 5.86, *p* = 0.02; F2(1,508) = 14.62, *p* < 0.001) showed (by grammaticality analysis) that participants reacted faster to number feature violations (593.66 ms) than to sentences containing their grammatical counterparts (720.83 ms) (F(1,46) = 70.21, *p* < 0.001). The analysis by feature revealed that participants reacted faster to sentences containing number violations (593.66 ms) than to sentences containing person violations (658.92 ms) (F(1,46) = 4.36, *p* = 0.042). A TYPE*GRAMMATICALITY*FEATURE interaction also emerged as (F1(1,48) = 4.72, *p* = 0.035; F2(1,508) = 2.97, *p* = 0.086) revealing that participants reacted faster to agent subject sentences containing number violations (582.97 ms) than to the corresponding grammatical sentences (748.19 ms) (F(1,46) = 54.49, *p* < 0.001). In sentences with patient subjects, participants reacted faster to ungrammatical sentences than to grammatical ones, regardless of whether the violation involved person (646.75 ms vs. 724.15 ms) or number feature manipulations (604.34 ms vs. 735.64 ms) (person: F(1,46) = 4.26, *p* = 0.045; number: F(1,46) = 4.26, *p* = 0.045). The analysis by type factor revealed no differences across type. The analysis by feature factor revealed that participants reacted faster to agent subject sentences containing number violations (671.08 ms) than to agent subject sentences containing person violations (582.97 ms) (F(1,46) = 7.23, *p* = 0.01).

### 3.2. Electrophysiological Results

**Figure 1:**
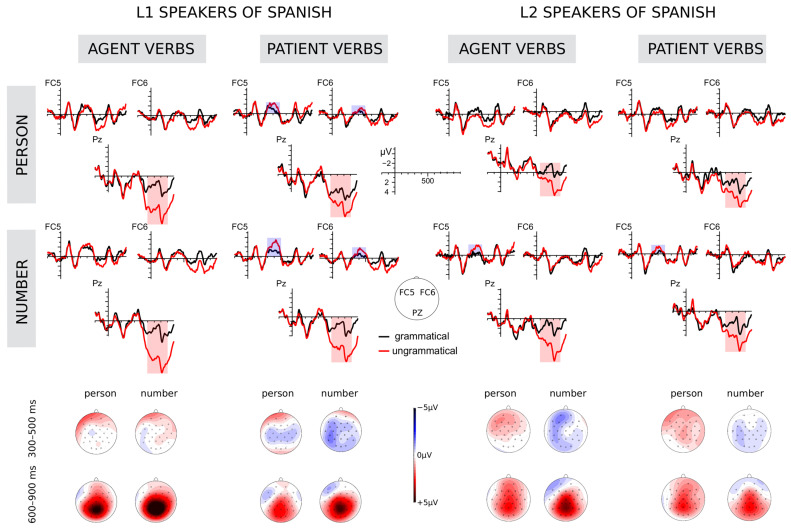
ERPs elicited at the critical word position in all conditions. Red lines represent the ungrammatical stimuli, while the black lines represent the grammatical stimuli. Significant differences between the grammaticality conditions are highlighted by the blue and red areas. Topographical amplitude difference maps for the grammaticality effect below were calculated as the average subtracting grammatical sentences from ungrammatical ones.

Regarding the early time window (300–500 ms) (see Figure 1), the analysis of lateral electrodes revealed a marginally significant main GROUP effect (F(1,46) = 3.8, *p* = 0.057) indicating a larger negativity for natives than non-natives (-0.67 µV vs. 0.27 µV) (see Table 5 for details). Besides, a main FEATURE effect emerged as well (F(1,46) = 6.53, *p* = 0.014), indicating a larger negativity for number feature than for person feature manipulations (−0.35 µV vs. −0.05 µV). Similarly, a FEATURE*GROUP interaction (F(1,46) = 7.84, *p* = 0.007) showed (analysis by feature) that verbs containing number feature manipulations generated a larger negativity than those containing person feature manipulations in native speakers (−0.99 µV vs. −0.35 µV), whereas no differences were found in non-natives (0.28 µV vs. 0.25 µV) (natives: F(1,23) = 14.33, *p* < 0.001; non-natives: F(1,23) = 0.03, *p* = 0.864). The comparison between both groups showed that natives elicited a larger negativity than non-natives in conditions involving number feature manipulations (−0.99 µV vs. 0.28 µV) (F(1,23) = 3.93, *p* = 0.016). Also, a TYPE*GRAM*GROUP interaction emerged (F(1,46) = 4.59, *p* = 0.037). The analysis by grammaticality factor showed that natives generated a larger negativity for patient subject violations than for grammatical patient subject sentences (−0.91 µV vs. −0.23 µV) (F(1,23) = 6.89, *p* = 0.012), whereas non-natives did not differ with regard to grammaticality neither in agent subject nor in patient subject sentences. The analysis by type factor showed that natives generated a larger negativity for grammatical agent subjects than for grammatical patient subjects (−0.84 µV vs. −0.23 µV) (F(1,23) = 5.77, *p* = 0.02), whereas non-natives did not differ with regard to the predicate type. Finally, further analyses showed that natives generated a larger negativity than non-natives for grammatical agent subject sentences (−0.84 µV vs. 0.37 µV) (F(1,23) = 3.7, *p* = 0.025), for patient subject violations (−0.91 µV vs. 0.21 µV) (F(1,23) = 1.59, *p* = 0.047), and marginally for agent subject violations (−0.71 µV vs. 0.31 µV) (F(1,23) = 0.73, *p* = 0.079). A FEATURE*GRAMMATICALITY interaction emerged (F(1,46) = 7.81, *p* = 0.008). The analysis by grammaticality factor showed no differences between grammatical and ungrammatical person manipulations, whereas number violations elicited a larger negativity than their grammatical counterparts (−0.6 µV vs. −0.11 µV) (F(1,46) = 9.17, *p* = 0.004). The analysis by feature factor revealed no differences between grammatical person and number, but number violations elicited a larger negativity than person violations (−0.6 µV vs. 0.51 µV) (F(1,46) = 13.43, *p* = 0.001). A FEATURE*GRAMMATICALITY*HEMISPHERE interaction also emerged (F(1,46) = 7.48, *p* = 0.009). Further analyses by grammaticality factor showed that sentences containing number violations generated a larger negativity than sentences containing grammatical number over the left hemisphere (−1,25 µV vs. −0.54 µV) (F(1,46) = 15.14, *p* < 0.001). The analysis by feature factor showed that number violations elicited a larger negativity than person violations over left and right hemisphere (−1,25 µV vs. −0.36 µV; 0.05 µV vs. 0.46 µV) (left: F(1,23) = 18.28, *p* < 0.001; right F(1,46) = 6.21, *p* = 0.016). Furthermore, a marginally significant FEATURE*GRAMMATICALITY*HEMISPHERE*REGION interaction emerged as well (F(2,92) = 3.23, *p* = 0.06).

The analysis of midline electrodes showed a main FEATURE effect (F(1,46) = 4.37, *p* = 0.042), indicating that number manipulations elicited a larger negativity than person manipulations (−0.41 µV vs. −0.06 µV). A FEATURE*GROUP interaction emerged as well (F(1,46) = 8.26, *p* = 0.006) and further analyses by feature factor showed a larger negativity for number manipulations than for person manipulations in native speakers (−1.21 µV vs. −0.37 µV), whereas no differences between number and person manipulations were observed in non-natives (0.39 µV vs. 0.25 µV) (natives: F(1,23) = 12.33, *p* = 0.001; non-natives: F(1,23) = 0.31, *p* = 0.582). The t-test showed that natives elicited a larger negativity for number feature manipulations than non-natives (-1.21 µV vs. 0.39 µV) (F(1,23) = 1.49, *p* = 0.029).

A marginally significant FEATURE*GRAMMATICALITY interaction emerged as well (F(1,46) = 3.28, *p* = 0.077). The subsequent analysis by grammaticality factor showed no differences between person feature violations and the corresponding grammatical sentences, and similarly, no differences between number feature violations and the corresponding grammatical sentences were found. The analysis by feature factor revealed no differences between grammatical verbs containing person and number manipulations, but number violations elicited a larger negativity than person violations (−0.53 µV vs. 0.15 µV) (F(1,46) = 6.9, *p* = 0.012). A GRAM*REGION*GROUP interaction (F(2,92) = 9.11, *p* < 0.001) revealed no differences between natives and non-natives driven by grammaticality or group factors.

Regarding the late time window (600-900 ms), the analysis carried out over the lateral electrodes revealed a primary GRAMMATICALITY effect (F(1,46) = 38.96, *p* < 0.001), indicating that the ungrammatical sentences (2.37 µV) elicited a larger positivity than the grammatical ones (0.97 µV). In addition, a TYPE*GRAMMATICALITY interaction emerged (F(1,46) = 7.21, *p* = 0.01). The analysis by grammaticality factor showed that agent subject violations (2.49 µV) elicited a larger positivity than their grammatical counterparts (0.8 µV) (F(1,46) = 45.94, *p* < 0.001), and similarly, patient subject violations (2.24 µV) elicited a larger positivity than their grammatical versions (1.15 µV) (F(1,46) = 18.5, *p* < 0.001). The analysis by type factor showed a marginally larger positivity for grammatical patient subject sentences (1.15 µV) as compared with grammatical agent subject sentences (0.8 µV) (F(1,23) = 3.41, *p* = 0.071). A GRAM*REGION*GROUP interaction (F(2,92) = 3.94, *p* = 0.043) showed (by grammaticality analysis) that regarding native speakers, the ungrammatical sentences elicited a larger positivity than the grammatical ones marginally over the frontal regions and significantly over the centro-parietal regions (frontal: F(1,46) = 3.26, *p* = 0.077; middle: F(1,46) = 29.98, *p* < 0.001; posterior: F(1,46) = 41.73, *p* < 0.001). Regarding non-native speakers, the ungrammatical sentences similarly elicited a marginally larger positivity than the grammatical ones over the frontal regions and significantly over the central and posterior regions (frontal: F(1,46) = 3.56, *p* =0.065; middle: F(1,46) = 9.28, *p* = 0.004; posterior: F(1,46) = 16.48, *p* < 0.001). Finally, the t-test also revealed no differences between both groups when comparing grammatical sentences over each region or when comparing ungrammatical sentences. The analysis of the FEATURE*GRAM*REGION interaction (F(2,92) = 4.31, *p* = 0.037) showed (by grammaticality factor) that sentences containing person violations elicited a larger positivity than the corresponding grammatical sentences (frontal: F(1,46) = 5.46, *p* =0.024; middle: F(1,46) = 17.95, *p* < 0.001; posterior: F(1,46) = 30.55, *p* < 0.001), and, similarly, number violations generated a larger positivity than the corresponding grammatical sentences marginally over the frontal electrodes and significantly over the central and posterior electrodes (frontal: F(1,46) = 2.91, *p* = 0.095; middle: F(1,46) = 30.16, *p* < 0.001; posterior: F(1,46) = 53.6, *p* < 0.001). The analysis by feature factor showed that grammatical verbs containing person manipulations elicited a larger positivity (1.24 µV) than grammatical verbs containing number manipulations (0.79 µV) significantly over the central electrodes (F(1,23) = 4.05, *p* = 0.05), and marginally over the posterior electrodes (1.83 µV vs. 1.43 µV) (F(1,23) = 3.44, *p* = 0.07).

The analysis of midline electrodes showed a main GRAMMATICALITY effect (F(1,46) = 61.24, *p* < 0.001), indicating that the ungrammatical sentences (1.29 µV) generated a larger positivity than the grammatical ones (3.79 µV). The analysis of a significant TYPE*GRAMMATICALITY interaction (F(1,46) = 7.04, *p* = 0.011) (by grammaticality) showed that agent subject violations (4.04 µV) elicited a larger positivity than the corresponding grammatical agent subject sentences (1.12 µV) (F(1,46) = 62.8, *p* < 0.001), and similarly, patient subject violations (3.54 µV) elicited a larger positivity than grammatical patient subject sentences (1.47 µV) (F(1,46) = 34.89, *p* <0.001). The analysis by type factor revealed a larger positivity for agent subject violations (4.04 µV) than for patient subject violations (3.54 µV) (F(1,23) = 4.32, *p* = 0.043). A marginally significant GRAM*REGION*GROUP interaction also emerged (F(2,92) = 3.19, *p* = 0.058). Further analyses by grammaticality factor showed that, regarding native speakers, the ungrammatical sentences elicited a larger positivity than the grammatical ones over all regions (frontal: F(1,46) = 25.55, *p* < 0.001; middle: F(1,46) = 30.86, *p* < 0.001; posterior: F(1,46) = 53.04, *p* < 0.001). Regarding non-native speakers, similarly, ungrammatical sentences elicited a marginally larger positivity than grammatical sentences over the frontal regions and significantly larger positivity over the central and posterior regions (frontal: F(1,46) = 13.77, *p* < 0.001; middle: F(1,46) = 19.68, *p* < 0.001; posterior: F(1,46) = 22.7, *p* < 0.001). No further differences between the groups were observed. A FEATURE*GRAM*REGION interaction (F(2,92) = 8.66, *p* = 0.001) (by grammaticality analysis) indicated that person violations elicited a larger positivity than the corresponding grammatical sentences (frontal: F(1,46) = 33.19, *p* < 0.001; middle: F(1,46) = 33.11, *p* < 0.001; posterior: F(1,46) = 44.14, *p* < 0.001), and similarly, number violations generated a larger positivity than the corresponding grammatical sentences (frontal: F(1,46) = 16.47, *p* < 0.001; middle: F(1,46) = 37.81, *p* < 0.001; posterior: F(1,46) = 63.14, *p* < 0.001). The analysis by feature factor showed that grammatical sentences involving person manipulations (2.43 µV) elicited a slightly larger positivity than grammatical sentences involving number manipulations (1.83 µV) over posterior electrodes (F(1,23) = 4.01, *p* = 0.051).

### 3.3. Summary of the Results

Regarding accuracy, non-native speakers were less accurate than natives, and overall, participants were more accurate with sentences containing agents than with sentences containing patients as subjects. They were also more accurate with sentences containing number feature manipulations than with sentences containing person feature manipulations. As regards reaction times, non-natives were generally slower than natives, and participants reacted faster to number feature violations than to person feature violations. With respect to the differences between the agent subject and patient subject predicates, electrophysiological results in the early time-window (300–500 ms) revealed a frontal negative component as a response to patient subject violations in the L1 group and no effect in the L2 speakers. No negativity was elicited by agent subject predicate violations in either group. In addition, natives displayed larger negativity for grammatical agent subject predicates than for grammatical patient subject predicates, while no such effect was observed in the non-native group. Regarding feature processing, number feature violations yielded a left-lateralized negativity among all speakers and this negativity was significantly larger than that elicited by person violations. With regard to the late time-window (600–900 ms), in comparison with the grammatical sentences, all ungrammatical sentences yielded a positive component (P600). Additionally, grammatical patient subject verbs generated a slightly larger positivity than grammatical agent subject verbs, and agent subject verb violations elicited a larger positivity than patient subject verb violations. Regarding phi-features, both person and number violations produced a larger positivity than their grammatical versions. Furthermore, grammatical sentences involving person manipulations elicited more positive responses than grammatical sentences containing the number feature over central and posterior electrodes.

## 4. Discussion

In the present study we investigated how native and early and highly proficient non-native speakers of Spanish process intransitive predicates and phi-features in order to determine to what extent non-native speakers can do this in a native-like way. In the subsequent sections we will first discuss the similarities and differences found between native and non-native speakers and the implications for the hypotheses considered previously. Next, we will interpret our data in light of the agent-first hypothesis and Feature Distinctness Hypothesis.

Regarding the first aspect, in general, similar effects and interactions were found for non-native speakers as compared with native speakers of Spanish, that is, differences between the processing of agent subject and patient subject predicates were observed, and differences between person and number phi-features emerged for both groups. More specifically, all participants were more accurate when judging grammatical phi-features than when reading sentences containing ungrammatical phi-features. Similarly, they reacted faster to number violations than to their grammatical counterparts. L2 speakers were overall slower and less accurate than L1 speakers when judging the grammaticality of the sentences. Since both natives and non-natives stated native-like or very high proficiency level of Spanish, the differences can be hardly accounted for by a lower competence of the non-native speakers as compared with natives. In any case, despite some behavioral differences, similar effects and interactions between predicate type and phi-features were observed in all participants at the electrophysiological level. More precisely, all speakers displayed left-lateralized negativity as a response to number violations, while no differences between ungrammatical and grammatical person features were observed at this stage of processing in either group. This indicates that for all speakers it was easier to detect number violations than person violations. In a similar vein, agent subject verb and patient subject verb agreement violations yielded larger positivity than their grammatical agreement versions in the late time window (600–900 ms). Likewise, grammatical patient subject verbs elicited a slightly larger positivity than grammatical agent subject verbs, whereas a larger positivity was obtained for agent subject verb violations than for patient subject verb violations in all participants. This suggests that patient subject predicates were costlier to process than agent subject predicates for both native and non-native speakers.

Regarding the electrophysiological differences between L1 and L2 speakers, in the 300–500 ms time window natives showed higher sensitivity towards patient subject vs. agent subject predicate distinction than non-natives, that is, they displayed larger negativity for patient subject agreement violations than for grammatical patient subject sentences, while no such effect was observed for agent subject agreement conditions. Conversely, non-native speakers showed no negativity as a response to patient subject or agent subject predicate violations at this stage of processing. In addition, natives also showed larger negativity for grammatical agent subject agreement than for grammatical patient subject agreement. Conversely, non-natives processed both types of predicates alike. The fact that they showed smaller effects than natives (or no negativity at all) when presented with SV agreement violations is not new. This type of response has also been observed for L2 speakers of Basque [33], with the authors suggesting (after [36]) that smaller or absent effects may be due to a reduced degree of automaticity in the activation of processing resources. A potential (although speculative) explanation for this pattern of results may be the influence of case morphology on the way SV agreement is built and processed. More precisely, Spanish is a nominative-accusative language where agent and theme/patient subjects are morphologically identical (unmarked), while Basque is an ergative language where agent subjects are marked and patient subjects are not. Previous findings [5,7] indicate that case alignment is an aspect that non-native speakers are particularly sensitive to and that, even at low AoA and high proficiency, they do not process it similarly to natives if it diverges from the L1. Given that the L1 of the non-native speakers tested in the present study is Basque, those participants may rely to a larger extent on case morphology than native speakers of Spanish do when processing verb agreement, and consequently, they show no negativity to either agent subject or patient subject agreement violations because they can no longer rely on the morphological cues, present in their L1 (Basque) but absent in the L2 (Spanish). This explanation is in line with the Language Distance Hypothesis (LDH) [7], which claims that differences between native and non-native speakers will emerge if non-shared grammatical phenomena are tested [1,2,5,7].

In relation to the agent-first hypothesis, our results show that both native and non-native speakers of Spanish process agent subject sentences with greater ease than patient subject sentences, as indicated by higher accuracy scores for sentences containing agents as subjects than for sentences containing patients as subjects. These findings are also supported by electrophysiological data: the positivity (P600) elicited by grammatical patient subject verbs was larger than that caused by grammatical agent subject, suggesting that the former are costlier to process than the latter. On the contrary, agent subject verb violations yielded larger positivity than patient subject verb violations suggesting that those structures are costlier to repair than those involving ungrammatical patient subject verb agreement. Our findings are also validated by previous experimental studies carried out in other (nominative-accusative) languages, where differences between agent subject and patient subject predicates were reported and larger processing costs for patient subjects than for agent subjects were found as well. Meltzer-Asscher et al. [37], for instance, examined native speakers of English by means of the fMRI method while processing transitive and intransitive agent subject verbs and intransitive patient subject verbs. The results revealed longer response times and larger activation of the left Inferior Frontal Gyrus (IFG) for patient subject verbs as compared with both transitive and intransitive agent subject verbs. Similarly, in a self-paced noncumulative moving-window experiment Dekydtspotter and Seo [38] tested L1 and L2 speakers of English while reading sentences containing agent subject and patient subject predicates and reported significantly greater loads after patient subject verbs than after agent subject verbs, indicating larger processing cost for the former than for the latter.

Despite different methodologies used in all these studies, our results are consistent with the main findings: intransitive agent subject verbs and patient subject verbs are processed differently, and in nominative-accusative languages patient subject verbs are costlier to process than agent subject ones. Although these differences between both types of predicates have been interpreted in terms of a syntactic movement [37], we believe that they can be also attributed to the general preference for interpreting the first unmarked argument of the sentence as an agent rather than a theme [18,19] (see also [8,17]). In other words, in nominative-accusative languages, in which all subjects are morphologically indistinguishable, a larger processing cost for patient subject verbs (unaccusatives) than for agent subject verbs (unergatives) would stem from a misinterpretation of the first (animate, human) argument as an agent instead of a theme and this would force the parser to reanalyze and revise the (wrongly) predicted structure at the verb position, leading to longer response times and an overall larger processing cost for patient subject sentences than for agent subject sentences.

In regard to the second aspect tested in the present study, namely, phi-feature processing, both L1 and L2 speakers showed faster reaction times for sentences containing number feature manipulations than for those containing person feature manipulations, and larger negativity for the number violations than for the grammatical number. Larger negativity was also observed for number violations than for person violations. Previous studies comparing person and number features in Spanish found some kind of negative component for person and number violations [23,24]. Person violations examined in the present experiment yielded no negativity at all, and though this outcome should be interpreted with caution (null effect), we tentatively attribute it to the type of materials used in our study. More precisely, sentences where the person feature was manipulated contained the 2nd person subjects, which participants may have initially considered vocatives, not an option for sentences containing number feature manipulations, which had 3rd person subjects. In that case, sentences containing person feature manipulations could initially leave more room for interpretation and even lead to a potential garden path, that is, sentences such as “*Tú, (…)*” ‘You, (…)’ could be also understood as “*Hey, you, (…)*” and disambiguated at the verb position. As a consequence, person manipulations would be more difficult to process than number manipulations. Indeed, in the later stage of processing (600–900 ms) a larger positivity was observed for sentences containing a grammatical person than for those containing a grammatical number, thus suggesting that our interpretation may be on the right track. On the other hand, the negativities obtained for number violations are similar to the those found in previous studies in Spanish [23,24]. In sum, these results support the first claim of the Feature Distinctness Hypothesis, indicating that person and number features are processed differently, but do not support the idea that the person is more salient than the number, since the negativity was only observed for number violations, and similar P600 was found for both person and number violations.

All in all, our data show that non-native speakers of Spanish process agent subject and patients subject predicates as well as person and number features differently. In that sense, non-native speakers are similar to native speakers, who also revealed agent subject vs. patient subject and person vs. number processing differences, indicating that native-like competence can be attained whenever non-divergent grammatical properties are at play.

## 5. Conclusions

In this study, we examined the processing of intransitive predicates and phi-features by native and non-native speakers of Spanish. Our results lend support for the agent-first hypotheses, as indicated by a larger processing cost for the patient subject sentences than for agent subject costs in both L1 and L2 speakers. In other words, our data can be accounted for by an initial assumption that all sentence-initial non-marked animate arguments are agents. Consequently, whenever that expectation is not met, as in the case of patient subject sentences in comparison to agent subject ones, a larger processing cost will be observed. Likewise, we provided additional evidence on phi-feature processing and showed that person and number features are not processed similarly. Lastly, this study further supports the idea that at high levels of proficiency and an early AoA, native-like processing is attainable as long as the properties are shared between the native and the non-native language.

## Figures and Tables

**Table 1:** The following seven-point scale was applied for measuring the relative use of language: 1 = I speak only Spanish, 2 = I speak mostly Spanish, 3 = I speak Spanish 75% of the time, 4 = I speak Basque and Spanish with similar frequency, 5 = I speak Basque 75% of the time, 6 = I speak mostly Basque, 7 = only Basque. Proficiency level was determined by using the following seven-point scale: 7 = native-like proficiency, 6 = high proficiency, 5 = full proficiency, 4 = working proficiency, 3 = limited proficiency, 2 = low proficiency, 1 = very low proficiency. SDs values are in parentheses.

	L1 Speakers of Spanish *n* = 24	L2 Speakers of Spanish *n* = 24
Age	20.5 (2.9)	21.8 (2.9)
AoA of Spanish	-	5.7 (1.9)
Sex (# males)	10	5
*Relative use of Spanish*		
Before primary school (0–3yrs)	1.56 (0.14)	6.75 (0.09)
*Primary school (4*–*12 yrs)*		
Home	1.22 (0.12)	6.63 (0.15)
School	4.3 (0.33)	6.58 (0.13)
Others	2.22 (0.25)	6.54 (0.12)
*Secondary school (12*–*18 yrs)*		
Home	1.3 (0.12)	6.63 (0.13)
School	3.74 (0.32)	6.08 (0.18)
Others	2.19 (0.23)	6 (0.16)
*At time of testing*		
Home	1.37 (0.18)	6.34 (0.22)
University/Work	3.74 (0.32)	5.58 (0.27)
Others	3.07 (0.29)	5.34 (0.25)
*Self-rated proficiency: Spanish*		
Comprehension	6.74 (0.09)	6.5 (0.16)
Speaking	6.74 (0.09)	6.04 (0.15)
Reading	6.67 (0.11)	6.54 (0.13)
Writing	6.6 (0.11)	5.71 (0.19)
*Self-rated proficiency: Basque*		
Comprehension	6.59 (0.1)	7 (0)
Speaking	6.07 (0.14)	6.92 (0.06)
Reading	6.52 (0.12)	6.92 (0.06)
Writing	6.3 (0.13)	6.75 (0.09)

Notes: # males: number of male participants.

**Table 2:** Sample of the materials used in the experiment.

Conditions			Sentence Examples
Subject Type	Feature	Grammaticality	
Agent	person	grammatical	(1) Tú, dentro de poco, **actuarás** en Hollywood.
			you, within a little, act.FUT.2SG in Hollywood
		ungrammatical	(2) Tú, dentro de poco, * **actuaré** en Hollywood.
			you, within a little, act.FUT.1SG in Hollywood
			“You will shortly play in Hollywood.”
	number	grammatical	(3) Él/Ella dentro de poco, **actuará** en Hollywood.
			he/she, within a little, act.FUT.3SG in Hollywood
		ungrammatical	(4) Él/ella, dentro de poco, **actuarán** en Hollywood.
			he/she, within a little, act.FUT.3PL in Hollywood
			“He/She will shortly play in Hollywood.”
Patient	person	grammatical	(5) Tú, lo antes posible, **vendrás** de visita.
			you, the earliest possible, come.FUT.2SG of visit
		ungrammatical	(6) Tú, lo antes posible, * **vendré** de visita.
			you, the earliest possible, come.FUT.1SG of visit
			“You will pay a visit as soon as possible.”
	number	grammatical	(7) Él/ella, lo antes posible, **vendrá** de visita.
			he/she, the earliest possible, come.FUT.3SG of visit
		ungrammatical	(8) Él/ella, lo antes posible, * **vendrán** de visita.
			he/she, the earliest possible, come.FUT.3PL of visit
			“He/she will pay a visit as soon as possible.”

NOTES: FUT.: future tense; 1,2,3: first, second and third person; SG: singular; PL: plural; *: the ungrammatical version of the word.

**Table 3:** Percentage of correct responses (%) and mean reaction times (ms) (SDE in parentheses).

	Accuracy in %				Response Times in milliseconds			
	Grammatical		Ungrammatical		Grammatical		Ungrammatical	
	Natives	Non-Natives	Natives	Non-Natives	Natives	Non-Natives	Natives	Non-Natives
Agent person	96.6 (1.2)	96.5 (1.1)	90.8 (1.3)	81.6 (4.1)	705.0 (58)	730.0 (56)	644.3 (55)	697.9 (60)
Agent number	95.3 (2.0)	92.3 (1.7)	91.4 (1.5)	89.5 (2.4)	711.4 (58)	785.0 (55)	576.2 (47)	589.8 (38)
Patient person	93.9 (1.6)	92.3 (1.5)	90.1 (0.9)	81.2 (4.3)	713.8 (54)	734.5 (57)	590.5 (46)	703.0 (66)
Patient number	95.1 (2.2)	92.4 (1.4)	92.7 (1.6)	87.2 (2.6)	718.2 (58)	753.1 (48)	576.1 (46)	632.6 (44)

**Table 4:** Statistical results (accuracy and response times). Notes: GROUP: type of participants (natives, non-natives); GRAM: grammaticality (grammatical, ungrammatical); TYPE: predicate type (agent subject verbs, patient subject verbs); FEAT: feature type (person, number); F1: analysis by subject; F2: analysis by item; ^a^ *p* < 0.1, * *p* < 0.05, *** *p* < 0.001.

	Accuracy		Response Times	
	F1 (1,46)	F2 (1,508)	F1 (1,46)	F2 (1,508)
GROUP	4.68 *	69.15 ***	0.57	3.98 *
GRAM	16.78 ***	131.6 ***	19.98 ***	201.64 ***
GRAM × GROUP	2.21	16.94 ***	0.19	0.03
TYPE	6.73 *	3.71 ^a^	0.05	0.01
TYPE × GROUP	1.57	1.9	0.49	0.01
FEAT	1.69	7.88 ***	1.75	0.84
FEAT × GROUP	0.46	2.34	0.06	<0.01
TYPE × GRAM	2.38	0.95	0.01	0.59
TYPE × GRAM × GROUP	0.52	0.37	3.6 ^a^	3.8 ^a^
GRAM × FEAT	5.22 *	30.89 ***	5.81 *	14.62 ***
FEAT × GRAM × GROUP	2.53	2.19	1.24	1.18
TYPE × GRAM × FEAT	3.31 ^a^	12.28 ***	4.72 *	2.97 ^a^
TYPE × FEAT × GRAM × GROUP	2.75	2.43	0.09	0.05

**Table 5:** Statistical results (EEG data) Notes: GRAM: grammaticality (two levels); TYPE: predicate type (two levels); FEAT: feature type (two levels); HEM: Hemisphere (two levels); REG: Anterior-Posterior factor (3 levels); df: degrees of freedom. ^a^ *p* < 0.1, * *p* < 0.05, ** *p* < 0.01, *** *p* < 0.001.

		300–500 ms		600–900 ms	
		Lateral	Midline	Lateral	Midline
	*df*	*F*	*F*	*F*	*F*
GROUP	1,46	3.8 ^a^	2.78	0.04	0.38
GRAM	1,46	1.36	0.29	*** 38.96	*** 61.24
GRAM × GROUP	1,46	1.1	0.52	1.81	1.68
TYPE	1,46	0.5	0.7	0.13	0.15
TYPE × GROUP	1,46	1.99	2	0.06	0.07
FEAT	1,46	* 6.53	* 4.37	0.65	0.09
FEAT × GROUP	1,46	** 7.84	** 8.26	0.65	1.13
TYPE × GRAM	1,46	2.74	0.54	* 7.21	* 7.04
TYPE × GRAM × GROUP	1,46	* 4.59	2.66	3.07 ^a^	1.9
FEAT × GRAM	1,46	** 7.81	3.28 ^a^	1.36	1.31
FEAT × GRAM × GROUP	1,46	3.33 ^a^	2.96 ^a^	2.2	1.22
TYPE × FEAT × GRAM	1,46	0.07	0.09	<0.01	0.01
TYPE × FEAT × GRAM × GROUP	1,46	0.21	0.12	0.13	0.35
GRAM × HEM	1,46	2.63	-	** 7.94	-
GRAM × HEM × GROUP	1,46	0.34	-	<0.01	-
TYPE × GRAM × HEM	1,46	<0.01	-	0.01	-
TYPE × GRAM × HEM × GROUP	1,46	0.01	-	0.08	-
FEAT × GRAM × HEM	1,46	** 7.48	-	0.44	-
FEAT × GRAM × HEM × GROUP	1,46	0.09	-	0.11	-
TYPE × FEAT × GRAM × HEM	1,46	1.19	-	0.94	-
TYPE × FEAT × GRAM × HEM × GROUP	1,46	1.19	-	<0.01	-
GRAM × REG	2,92	3.65	*** 10.21	*** 36.19	*** 38.83
GRAM × REG × GROUP	2,92	0.77	*** 9.59	* 3.94	3.19 ª
TYPE × GRAM × REG	2,92	0.71	0.77	0.26	1.57
TYPE × GRAM × REG × GROUP	2,92	0.01	0.25	0.06	0.22
FEAT × GRAM × REG	2,92	1.4	1.5	* 4.31	** 8.66
FEAT × GRAM × REG × GROUP	2,92	1.42	0.15	2.05	0.09
TYPE × FEAT × GRAM × REG	2,92	0.83	0.59	0.3	0.07
TYPE × FEAT × GRAM × REG × GROUP	2,92	0.07	0.74	0.02	0.66
GRAM × HEM × REG	2,92	0.44	-	*** 9.53	-
GRAM × HEM × REG × GROUP	2,92	0.15	-	1.47	-
TYPE × GRAM × HEM × REG	2,92	1.24	-	0.25	-
TYPE × GRAM × HEM × REG × GROUP	2,92	0.6	-	2.01	-
FEAT × GRAM × HEM × REG	2,92	3.23	-	0.04	-
FEAT × GRAM × HEM × REG × GROUP	2,92	1.11	-	0.71	-
TYPE × FEAT × GRAM × HEM × REG	2,92	0.12	-	0.25	-
TYPE × FEAT × GRAM × HEM × REG × GROUP	2,92	0.25	-	0.27	-

## Data Availability

Derived data supporting the findings of this study are available from the corresponding author upon request.
